# A 3′UTR polymorphism disrupts IRF2BP2 autoregulation through an eIF4H translational enhancer

**DOI:** 10.3389/fgene.2026.1846555

**Published:** 2026-05-28

**Authors:** An Duong, Hsiao-Huei Chen, Alexandre F. R. Stewart

**Affiliations:** 1 Department of Biochemistry, Microbiology and Immunology, University of Ottawa, Ottawa, ON, Canada; 2 University of Ottawa Heart Institute, Ottawa, ON, Canada; 3 Centre for Infection, Immunity and Inflammation, University of Ottawa, Ottawa, ON, Canada; 4 Ottawa Hospital Research Institute, Ottawa, ON, Canada; 5 Department of Medicine, University of Ottawa, Ottawa, ON, Canada; 6 Department of Cellular and Molecular Medicine, University of Ottawa, Ottawa, ON, Canada

**Keywords:** eukaryotic translation initiation factor 4H, genetic polymorphism, interferon regulatory factor (IRF), macrophage, translation regulation

## Abstract

**Introduction:**

Interferon regulatory factor 2 binding protein 2 (IRF2BP2) suppresses the interferon response and inflammation. Individuals who carry 2 copies of a genetic variant (rs3045215) that deletes 9 nucleotides from the long 3′UTR of IRF2BP2 have lower IRF2BP2 protein expression in white blood cells and increased risk of coronary atherosclerosis and calcification.

**Methods and Results:**

RNAfold revealed that the deletion variant of IRF2BP2 disrupts an RNA stem-loop structure that can recruit the eukaryotic initiation factor 4H (eIF4H) to facilitate translation. siRNA knockdown of eIF4H reduced expression of endogenous IRF2BP2 protein. Similarly, it impaired translation of a luciferase reporter bearing the whole 3′UTR of IRF2BP2 but had no effect on one bearing the 9-nucleotide deletion variant (rs3045215). This deletion variant happens to be co-inherited with an IRF2BP2 coding variant that changes proline to serine at position 78. Overexpression of either isoform of IRF2BP2 (Pro^78^ or Ser^78^) suppressed translation of the luciferase reporter containing the whole IRF2BP2 3′UTR to the same level but had no effect on the deletion-bearing reporter. RNA gel mobility shift assay using cytosolic extracts of LPS-stimulated THP1 macrophages revealed that the 9-nucleotide deletion variant prevents endogenous IRF2BP2 protein from interacting with its own 3′UTR RNA sequences.

**Conclusion:**

The rs3045215 9-nucleotide deletion that increases the risk of heart disease abolishes IRF2BP2 autoregulation through an eIF4H-dependent translational enhancer.

## 1 Introduction

Interferon regulatory factor 2-binding proteins (IRF2BP) encode a family of transcriptional regulators including IRF2BP1, IRF2BP2 and IRF2BPL. Each member bears a conserved N-terminal C4 zinc finger domain, a nuclear localization signal and a C-terminal CH3C4 RING domain. IRF2BP2 was the first family member described by Childs and Goodbourn (2003) as a transcriptional co-repressor that interacts with IRF2 through its RING domain to inhibit transcription of interferon-inducible genes (Childs and Goudbourn, 2003). The IRF2BP factors have non-redundant functions; IRF2BP1 not only represses IRF2-dependent gene expression (Childs and Goudbourn, 2003) but also interacts and competes with the TGF-beta-induced homeodomain factor (TGIF) to enhance TGFβ signaling (Faresse et al., 2008). IRF2BPL plays a crucial role in neurodevelopment and loss of IRF2BPL causes a severe neuro-regressive disorder due to excessive WNT signaling in neurons (Marcogliese et al., 2022).

IRF2BP2 also plays an important role in several IRF2-independent pathways, including cell survival and protection from apoptosis by repressing p53-mediated transactivation of the apoptosis regulator BAX (Koeppel et al., 2009). IRF2BP2 also represses NFAT1-mediated gene expression in Jurkat T cells (Carneiro et al., 2011), YAP/TEAD4 signalling in hepatocytes (Feng et al., 2020) and the JAK/STAT signaling pathway, since STAT1 is activated in peripheral lymphocytes of individuals with truncation mutations in IRF2BP2 (Palmroth et al., 2021). While the preponderance of genes affected by ablation of Irf2bp2 are upregulated in the liver, skeletal muscle and heart of Irf2bp2 null mice, suggesting a co-repressor function of IRF2BP2, it may also function as a co-activator of Sirt1, Taf4 and Nkx2-3 (Vilmundarson et al., 2022). We identified IRF2BP2 as a partner of VGLL4 in a yeast two-hybrid screen of the human heart and found that it activates a vascular endothelial growth factor A promoter when co-expressed with TEAD4 and VGLL4 (Teng et al., 2010). IRF2BP2 was also reported to act as a strong co-activator of the glucocorticoid receptor (GR) and to modulate GR and tumor necrosis factor alpha (TNF) signaling in lung epithelial A549 cells (Manjur et al., 2019).

Congenital mutations in IRF2BP2 have been tied to common variable immunodeficiency (CVID) (Keller et al., 2016; Udemgba et al., 2025). In a recent survey of 34 individuals with mutations in IRF2BP2 from 18 different families, 31 (91%) displayed CVID, 22 (65%) had gastrointestinal diseases (chronic abdominal pain, inflammatory bowel disease, Crohn’s disease, and irritable bowel syndrome) and the third most common diagnosis was inflammation in 19 individuals (56%). Somatic loss-of-function mutations have been identified in cases of lymphoma (Bruno et al., 2014; Mazharuddin et al., 2018) and acute promyelocytic leukemia (Alotaibi et al., 2020). In mice, deletion of Irf2bp2 is lethal due to the loss of an essential function related to fetal myeloid-dependent hepatic hematopoiesis (Stadhouders et al., 2015). However, Irf2bp2-null mice that acquire wild type myeloid progenitors from older male siblings through trans-maternal microchimerism are rescued from fetal lethality, but succumb to lymphoma as adults (Vilmundarson et al., 2022). Ablation of *Irf2bp2* in macrophages augments their inflammatory response, increases the susceptibility of mice to atherogenic mutations (Chen et al., 2015) and impairs recovery from ischemic brain injury (Cruz et al., 2017). These studies suggest that reduced IRF2BP2 expression may enable chronic inflammation.

Common genetic variants in the vicinity of IRF2BP2 affect traits related to heart disease, including elevated LDL cholesterol (rs10910476) (Richardson et al., 2020) and diastolic blood pressure (rs1329128), as well as inflammatory bowel disease (rs10910476) (Liu et al., 2023) and osteoporosis (rs6672925) (Morris et al., 2019), known risks of coronary artery disease (Laroche et al., 2017; Guo et al., 2025). Our lab has identified a 9-nucleotide deletion polymorphism (rs3045215) that works by a recessive model to increase the risk of coronary artery atherosclerosis (Chen et al., 2015) and coronary artery calcification in men (Vilmundarson et al., 2021). In individuals homozygous for the deletion, the level of IRF2BP2 protein in their peripheral blood mononuclear cells is reduced (Chen et al., 2015).

Activity studies of luciferase reporters bearing the entire 3′ UTR of IRF2BP2 (2,868 nucleotides) but differing only in the 9 nucleotide deletion revealed a translational enhancer at the 3′ UTR whose function is disrupted by the deletion (Chen et al., 2015). Further, our studies show that this deletion variant does not affect the rate of mRNA decay following actinomycin-D treatment to block transcription (Chen et al., 2015). This IRF2BP2 3′UTR translational enhancer functions in part by excluding the binding of a translation inhibitor ELAVL1 (Vilmundarson et al., 2021). On the other hand, recruitment of the eukaryotic initiation factor 4H (eIF4H) to RNA stem-loop structures to facilitate the RNA helicase function of eIF4A is particularly important to enhance translation of GC-rich RNA sequences that are difficult to unwind for translation (Marintchev et al., 2009; Sun et al., 2012). The IRF2BP2 mRNA has an extremely GC-rich (>80%) sequence in the 5′UTR spanning 684 nucleotides that is likely difficult to unwind and translate without a translational enhancer. Here, we report that the translational enhancer of the IRF2BP2 3′UTR disrupted by the rs3045215 deletion variant relies on eIF4H to augment translation. Furthermore, our studies show that IRF2BP2 targets this enhancer region to suppress and regulate its own expression.

## 2 Materials and methods

### 2.1 IRF2BP2 expression plasmids

Genomic DNA obtained from the Ottawa Heart Genomics Study (Dandona et al., 2010) was genotyped using PCR primers (forward: 5′-CTT CAC CGA ACC CGT CTG-3′, reverse: 5′- CTG CTG CCG AAG TCA GAG-3′) to identify samples containing either variant of rs7545855. The entire coding sequence of IRF2BP2 containing either variant was then cloned into a pCDEF3 backbone to produce pCDEF3-IRF2BP2-Ser^78^ and pCDEF3-IRF2BP2-Pro^78^ and verified by Sanger sequencing (Applied Biosystems 3730DNA Analyzer, The Ottawa Hospital DNA Sequencing Facility).

### 2.2 RNA folding

The 3′ UTR sequence of IRF2BP2 100 nucleotides on either side of the rs3045215 indel variant was subjected to RNA folding using the RNAfold webserver (http://rna.tbi.univie.ac.at/cgi-bin/RNAWebSuite/RNAfold.cgi). The minimum free energy algorithm was used to predict RNA secondary structure plots with reliability annotations.

### 2.3 Luciferase reporter assays

The IRF2BP2 3′UTR luciferase reporter constructs were previously described (Chen et al., 2015). Genomic DNA obtained from the Ottawa Heart Genomics Study was genotyped to identify samples homozygous for either allele of rs7545855. IRF2BP2 cDNA of each allele was then cloned into a pCDEF3 expression plasmid (Addgene plasmid #49212) and verified by Sanger sequencing (Applied Biosystems 3730DNA Analyzer, The Ottawa Hospital DNA Sequencing Facility).

HEK293 cells were seeded in 12-well plates (5 × 10^5^ cells/mL) and allowed to adhere overnight. The following day, cells were transfected in triplicate using Lipofectamine 3,000 (Invitrogen) according to the manufacturer’s protocol with 0.5 μg/mL of either variant of the IRF2BP2 3′UTR luciferase assay. 24 h after transfection, cells were harvested and the lysate was extracted. Luminescence readings were measured using a Berthold Technologies FB12 Luminometer.

### 2.4 siRNA assay

HEK293 cells were plated and transfected as described above. Cells were co-transfected with 16 nM of control (Santa Cruz Biotechnology sc-37007) or eIF4H siRNA (Santa Cruz Biotechnology sc-89585) according to the manufacturer’s protocol. The eIF4H siRNA cocktail consists of 3 pooled siRNA duplexes (sc-89585A, 5′-GCU​UUG​CUG​UAU​CUA​UCU​Att-3’; sc-89585B, 5′- GCA​GAG​ACC​UUG​UUG​GUA​Utt-3′; sc-89585C, 5′ GUA​ACA​CCA​ACA​CUU​AAC​Utt-3′; sense strands). 24 h after transfection, cell lysates were extracted and luminescence readings measured as described above.

### 2.5 Immunoblots

HEK293 cells were transfected with 250 ng/mL of control or eIF4H siRNA and protein was extracted 24 h later in RIPA buffer containing protease inhibitors (Roche–cOmplete Protease Inhibitor Tablets, ThermoFisher). Proteins were size fractionated by tris-glycine extended TGX (4%–15%) polyacrylamide gel (Bio-Rad) electrophoresis followed by electroblotting to polyvinylidene fluoride (PVDF) membranes (0.2 µm) for 1.5 h (Thermo Scientific). Proteins were visualized with the following antibodies: anti-eIF4H rabbit polyclonal antibody (Cell Signaling Technology #2444S) used at a dilution of 1:1,000 in 5% non-fat milk with TBST (Tris-buffered saline Tween, 20 mM Tris, 0.1% Tween 20) overnight at 4 °C; anti-IRF2BP2 antibody (Invitrogen #PA5100580) at a dilution of 1:700 in 5% non-fat milk with TBST overnight at 4 °C; anti-GAPDH mouse monoclonal antibody (R&D Systems #MAB5718) at a dilution of 1:10,000 in 5% non-fat milk with TBST for 1 h at room temperature; goat anti-rabbit IgG (Life Technologies #31460) used at a dilution of 1:10,000 in 5% non-fat milk with TBST for 1 h at room temperature; goat anti-mouse IgG (R&D Systems #HAF007) used at a dilution of 1:10,000 in 5% non-fat milk with TBST for 1 h at room temperature. Bands were revealed by chemiluminescence (Bio-Rad Clarity Max ECL Western blot Substrate, #1705062) and were quantified using ImageJ (https://imagej.net/ij/).

### 2.6 RNA electrophoresis mobility shift assay

The RNA electrophoresis mobility shift assay was used to determine whether proteins bind differentially to RNA probes containing the non-deletion and deletion variants of rs3045215, as described previously (Vilmundarson et al., 2021). Non-deletion probe: 5′-UAG​GCA​CUU​UAUU​AUA​ACUGGA​AUU​UGA​C-3′; Deletion probe: 5′-UAG​GCA​CUU​UGG​AAU​UUG​AC-3″ (Integrated DNA Technologies). RNA probes were 5′-end labeled using Gamma P-32 ATP and T4 Polynucleotide Kinase at 37 °C for 1 h. The reaction was stopped using 0.5 M EDTA (pH 8.0) and then the samples were purified using RNase-free G-25 Sephadex spin columns (Roche) and 50,000 CPM were used per well. The following solutions were prepared for non-deletion and deletion probes, separately: 10X binding buffer, 1µL; radiolabeled probe (50,000 CPM dilution), 1 μL; 50% glycerol, 1.5 µL; 1 M DTT, 0.2 µL; RNasin (Promega), 0.2 µL; Yeast Total RNA (10 mg/mol), 0.2 µL; 5% xylene cyanol, 1 μL; DEPC-treated ddH_2_O, 2.9 µL. All components were prepared or purchased RNase-free. Solutions were added to protein extracts, gently vortexed, spun down and incubated at 37 °C for 3 min. THP1 cells were cultured as described previously (Vilmundarson et al., 2021) and treated with PBS or LPS (100 ng/mL) prior to cytoplasmic protein extraction in lysis buffer (1% Triton, 25 mM Tris HCl, pH 7.4, 40 mM KCl) according to the method of Fillebeen et al. (2014). Antibodies for gel shift interference assays were a rabbit anti-human IRF2BP2 peptide-specific antibody (Teng et al., 2010) and a rabbit polyclonal anti-AUF1 antibody (#ab50692, Abcam).

### 2.7 IRF2BP2 3′UTR luciferase reporters

The IRF2BP2 3′UTR luciferase reporter construct was previously described (Chen et al., 2015). Briefly, PCR primers containing an XbaI site (underlined) (forward: 5′-CCT CTA GAG CAC ACA TGC AGA AAT GCA GAG TC-3′, reverse: 5′-CCT CTA GAC ATA CAG ATA GCT ACC ACA AAT TAG GTC-3′) were used to amplify the complete 2,877 nucleotide long fragment of the 3′UTR of human IRF2BP2 with or without the 9-nucleotide deletion of rs3045215. The fragments were cloned into a CMV enhancer-driven pGL3 luciferase vector.

### 2.8 Statistical analysis

Raw luciferase activity units were normalized to empty vector control values and averaged. Two-way ANOVA was used to analyze groups with two or more variables and *post hoc* pairwise comparisons used Tukey’s test to correct for multiple comparisons. All statistical tests were carried out using the GraphPad (ver. 9.0) software.

## 3 Results

### 3.1 The 9-nucleotide rs3045215 deletion disrupts a stem-loop structure in the 3′UTR of IRF2BP2

Our previous work established that the rs3045215 deletion variant increased risk of coronary atherosclerosis and calcification and patients who carry two copies of this deletion variant have lower IRF2BP2 protein levels in their circulating white blood cells.

**FIGURE 1:**
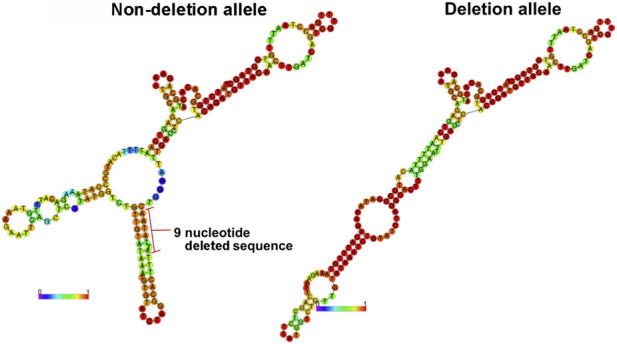
Predicted RNA structure of IRF2BP2 3′UTR comparing the deletion (risk) and non-deletion (non-risk) variants (RNAfold Web Server). RNA secondary structure blot based on the minimum free energy and partition function. Scale bar shows probability. For based paired regions, color denotes base-pairing probabilities. For unpaired regions, the color denotes the probability of being unpaired.

This deletion variant reduces activity of a luciferase reporter bearing the entire 3′UTR sequence of IRF2BP2 but does not affect mRNA stability (Chen et al., 2015), indicating that the deletion variant reduces translation of IRF2BP2 mRNA. Complementary sequences in RNA can fold to form various stem-loop structures that sterically hinder the efficiency of translation unless they are recognized by RNA-binding proteins that relieve this hindrance. The RNAfold minimum-free-energy algorithm predicts the 3′UTR RNA of IRF2BP2 forms a stem-loop structure disrupted by the 9-nucleotide deletion of the rs3045215 as in the deletion variant (Figure 1).

The eukaryotic translation initiation factor 4H (eIF4H) is known to facilitate translation of mRNAs with complex 5′UTR sequences by recognizing stem-loop structures and promoting RNA unwinding by the eIF4A RNA helicase (Sun et al., 2012). While this function has been ascribed to an interaction of eIF4H with 5′UTR sequences, some initiation factors like eIF3 were found to interact mainly with 3′UTR sequences of highly translated mRNAs (Mestre-Fos et al., 2025). Furthermore, in an unbiased screen of protein-protein interactions, eIF4H was found to physically interact with IRF2BP2 (Wan et al., 2015), although the protein domains that mediate this interaction remain to be elucidated. For these reasons, we chose eIF4H as a candidate to test whether eIF4H may also regulate the IRF2BP2 translation by interacting with the 3′UTR stem-loop, and whether this regulation is affected by the rs3045215 deletion variant.

### 3.2 eIF4H promotes translation via the IRF2BP2 3′UTR, an effect abolished by the 9-nucleotide deletion of the rs3045215 variant

**FIGURE 2:**
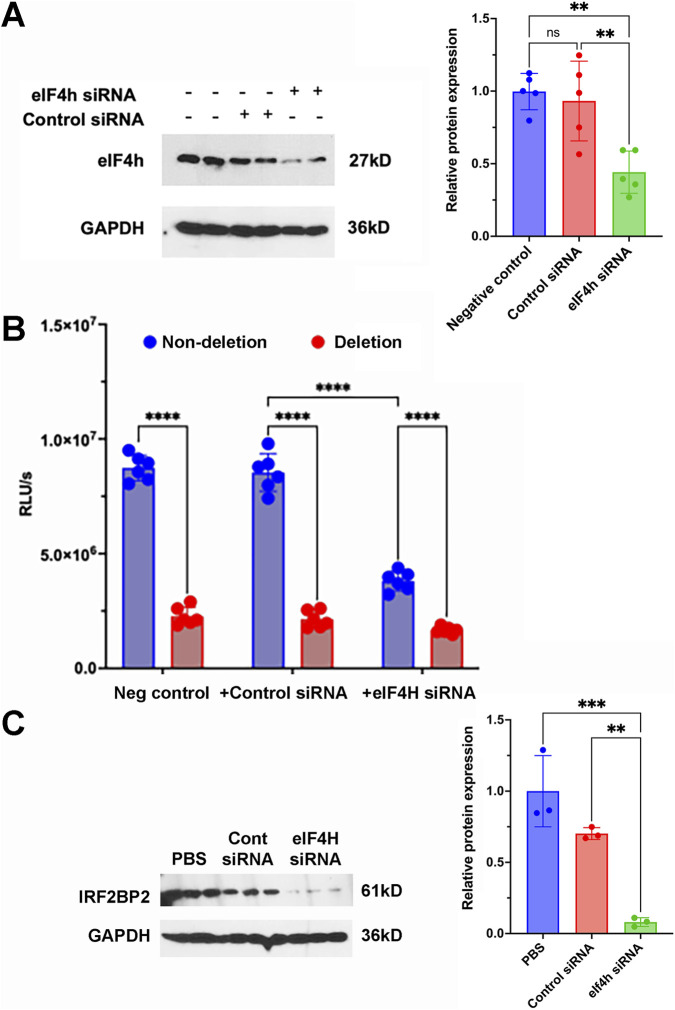
eIF4H is required for expression of the IRF2BP2 3′UTR non-deletion variant. **(A)** eIF4H siRNA lowers eIF4H protein expression in HEK293 cells. **, p < 0.01 by t-test, n = 3. **(B)** eIF4H siRNA lowers expression of the IRF2BP2 3′UTR luciferase reporter bearing the non-deletion but not the deletion variant. Luciferase reporter activities were expressed as fold of the non-deletion luciferase reporter activity at baseline. HEK293 cells were co-transfected with 250 ng/mL of either IRF2BP2 3′UTR variant luciferase reporter with eIF4H siRNA or control siRNA eIF4H (n = 6, ****, p < 0.0001). **(C)** Endogenous IRF2BP2 protein expression is reduced by eIF4H siRNA. (n = 3, ***, p < 0.001, **, p < 0.01 by t-test). GAPDH was used as a loading control.

To test whether eIF4H acts at the 3′UTR to regulates IRF2BP2 translation, HEK293 cells were co-transfected with an eIF4H siRNA or a control siRNA together with a firefly luciferase reporter carrying either rs3045215 allele (non-deletion or 9-nucleotide deletion) of the IRF2BP2 3′UTR. Of note, we did not use a second reporter plasmid to determine differences in transfection efficiencies as this reporter plasmid could also be affected by eIF4H siRNA, rendering it an unreliable control. Luciferase reporter activities were expressed as a fold of the non-deletion luciferase reporter activity at baseline for each experiment. Knockdown of eIF4H was confirmed by immunoblot (Figure 2A). In the presence of the eIF4H siRNA, luciferase activity of the non-deletion variant was reduced compared to the control siRNA. In contrast, the luciferase activity of the reporter carrying the deletion variant was not affected by the eIF4H siRNA (Figure 2B). Thus, eIF4H promotes translation of a reporter bearing the whole IRF2BP2 3′UTR-containing RNA sequence while this translational enhancement is lost if the 9-nucleotides of the rs3045215 variant are deleted.

Endogenous IRF2BP2 protein expression was also markedly reduced in HEK293 cells 24 h after eIF4H siRNA transfection (Figure 2C), while expression of GAPDH was not affected. These results are consistent with eIF4H having minimal effect on GAPDH expression, as reported previously (Vaysse et al., 2015), while maintaining high levels of IRF2BP2 translation *in vivo*.

### 3.3 The rs3045215 deletion variant disrupts IRF2BP2-dependent self-regulation

**FIGURE 3:**
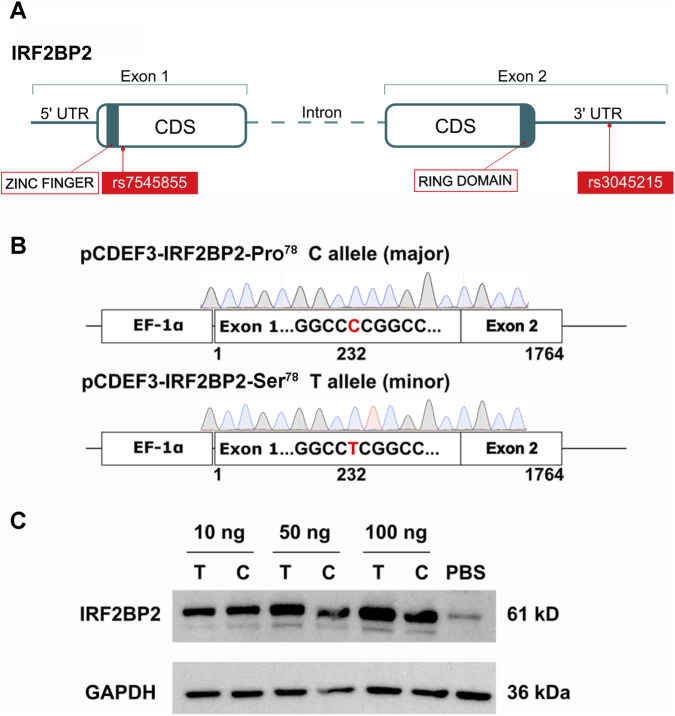
IRF2BP2 Ser^78^ variant in exon 1 is co-inherited with the 9-nucleotide deletion (rs3045125) in exon 2. **(A)** A schematic representation of IRF2BP2. A SNP variant in the first exon located just outside of the zinc finger domain (rs7545855) is co-inherited with a 9-nucleotide deletion variant in the 3′UTR (rs3045215). **(B)** An EF-1α-driven pCDEF3 expression plasmid carrying the coding sequence of IRF2BP2 with either the Pro^78^ C allele or the Ser^78^ T allele variant was created and confirmed by sequencing (electropherograms overlaid on the diagrams). **(C)** Transient transfection of expression plasmids (ng) to HEK293 cells produced similar levels of expression for each isoform, revealed by immunoblot. GAPDH was used as a loading control. PBS, phosphate-buffered saline, shows endogenous levels of IRF2BP2.

Given that eIF4H is required to mediate the translational enhancer function disrupted by the rs3045215 deletion variant and that eIF4H physically interacts with IRF2BP2 protein, we wanted to know how IRF2BP2 affects eIF4H-mediated translation enhancement. It should be noted that there are two coding variants of IRF2BP2 where a proline residue at position 78 is substituted for a serine residue in the first exon of IRF2BP2. Remarkedly, this Ser^78^ coding variant (rs7545855) is co-inherited with the 3′UTR 9-nucleotide deletion variant at rs3045215 (Figure 3A). We next tested whether over-expression of either IRF2BP2 protein variant would affect a luciferase reporter bearing the IRF2BP2 3′UTR sequence containing either the deletion or non-deletion variants.

**FIGURE 4:**
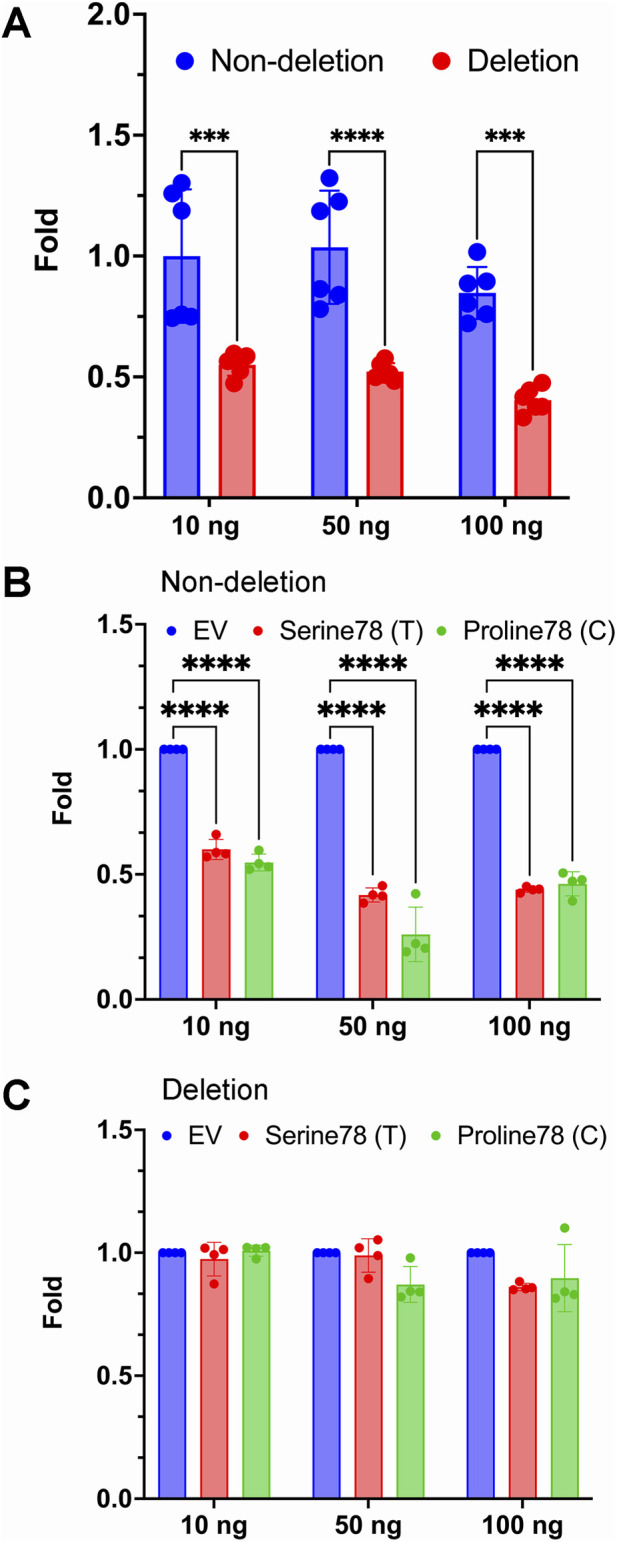
Both coding isoforms of IRF2BP2 suppress expression of the IRF2BP2 3′UTR luciferase reporter bearing the non-deletion rs3045215 allele but not the deletion allele. **(A)** The CMV-driven luciferase reporters carrying the 3′UTR sequence of IRF2BP2 with the deletion (risk; pCMV-Δ-3′UTR-IRF2BP2) allele is less active than the non-deletion (non-risk; pCMV-i-3′UTR-IRF2BP2) allele (n = 6, ***, *p < 0.0005*, ****, *p < 0.0001*). HEK293 were transfected with 250 ng/mL of either the non-deletion **(B)** or deletion **(C)** pCMV-3′UTR-IRF2BP2 luciferase reporters with increasing concentrations of empty vector (EV), pCDEF3-IRF2BP2-Pro^78^ or pCDEF3-IRF2BP2-Ser^78^ expression plasmids (10, 50 and 100 ng/mL, n = 6, ****, *p < 0.0001*). Luciferase reporter activities were expressed as fold of the non-deletion luciferase reporter activity at baseline.

To this end, we generated expression plasmids for each isoform (Figure 3B) and confirmed that similar levels of IRF2BP2 expression were achieved with 10, 50 and 100 ng of each expression plasmid (Figure 3C); this result suggests that the two isoforms have similar protein stabilities. Co-expression of either the Pro^78^ or Ser^78^ IRF2BP2 isoform markedly suppressed the luciferase activity of the non-deletion-bearing reporter to half the levels observed with empty vector (Figures 4A,B). Conversely, neither isoform of IRF2BP2 affected the expression of the 3′UTR luciferase reporter bearing the deletion variant of rs3045215 (Figure 4C). These luciferase reporter studies indicate that IRF2BP2 protein, regardless of isoform, regulates its own expression via its 3′UTR, an auto-regulatory function that is lost with the rs3045215 deletion variant.

### 3.4 The rs3045215 deletion variant disrupts IRF2BP2 interaction to its own 3′UTR sequence

**FIGURE 5:**
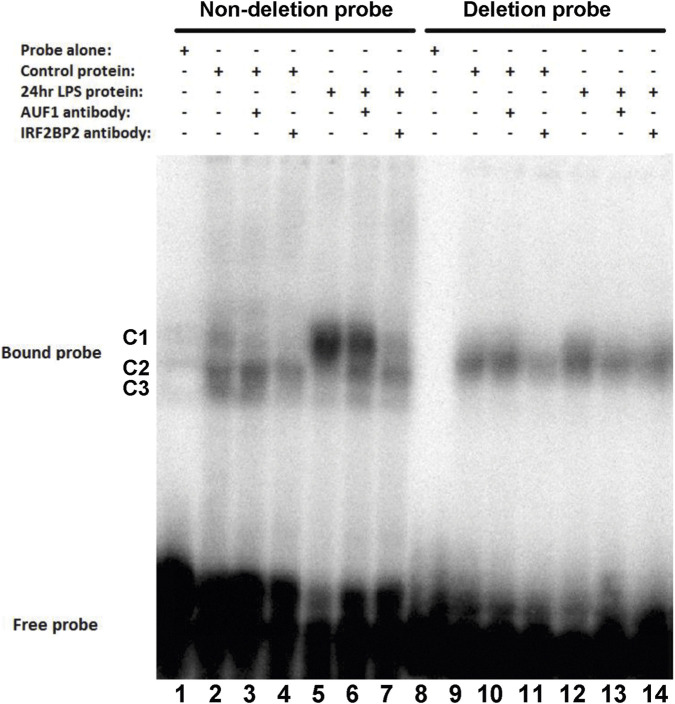
RNA gel mobility shift reveals specific binding of IRF2BP2 to rs3045215 non-deletion probe in cytoplasmic extract of LPS-treated THP1 cells. The non-deletion probe produced 3 shifted complexes (C1, C2, and C3) whereas the deletion probe produced a single shifted complex. Complex C1 contains IRF2BP2 protein, as revealed by antibody interference tested with AUF1 and IRF2BP2 antibodies. Free probe (not bound to protein), bound probe (bound to protein).

Since IRF2BP2 suppresses its own expression through a 3′UTR sequence disrupted by the rs3045215 deletion, we next asked whether IRF2BP2 could form a complex with RNA sequences in the vicinity of the rs3045215 deletion. RNA gel shift using RNA probes of 29 nucleotides (containing the rs3045215 non-deletion variant) or 20 nucleotides (containing the rs3045215 deletion variant) was carried out with cytosolic extracts of THP1 human monocyte cells. Differential binding to these RNA probes was observed, with 3 complexes (C1, C2 and C3) formed by the probe containing the non-deletion (Figure 5, lanes 1–7) whereas a single complex was formed by the deletion probe (Figure 5, lanes 8–14). LPS treatment of THP1 cells, a condition that suppresses IRF2BP2 expression (Chen et al., 2015; Vilmundarson et al., 2021), was associated with increased formation of complex (C1). To identify the protein associated with this C1 RNA-protein complex, antibodies to different protein candidates, including AUF1 and IRF2BP2, were tested. Addition of the AUF1 antibody had no effect on complex C1 formation, whereas addition of an antibody to IRF2BP2 interfered with the formation of the C1 complex (Figure 5, compare lanes 6 and 7), indicating that this effect is specific. These results indicate that IRF2BP2, a factor predominantly found in the nucleus, can also be present in the cytoplasm. Further, IRF2BP2 is part of the C1 complex whose formation is increased by LPS, likely by increasing the cytosolic levels of endogenous IRF2BP2 or by increasing the binding affinity of cytosolic IRF2BP2 to its 3′UTR or an RNA-binding protein.

**FIGURE 6:**
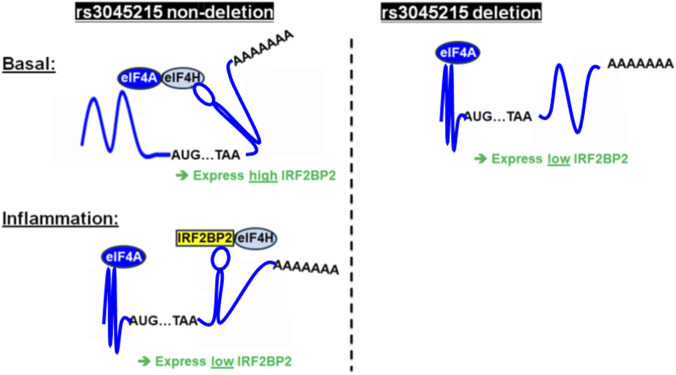
Model accounting for low expression of the rs3045215 deletion variant. The reference allele of rs3045215 contains a stem loop structure that interacts with eIF4H to facilitate eIF4A-dependent translation. Inflammation (as occurs with LPS challenge) recruits IRF2BP2 (Pro^78^) to its own 3′UTR displacing eIF4H. Of note, THP1 cells are homozygous for the Pro^78^ variant (Vilmundarson et al., 2021). The deletion variant is a loss of function recessive allele that disrupts the eIF4H-dependent translational enhancer, and IRF2BP2-mediated autoregulation, resulting in low levels of IRF2BP2 protein as seen for the non-deletion variant during inflammation.

Taken together, our studies suggest that IRF2BP2 protein levels are regulated by its 3′UTR sequence through an eIF4H-dependent mechanism that is disrupted by 9-nucleotide deletion in the rs3045215 variant. Furthermore, our studies suggest that IRF2BP2 exploits this mechanism to regulate its own expression under inflammatory conditions; as summarized in a model (Figure 6).

## 4 Discussion

IRF2BP2 in macrophages works as a key regulator of innate immunity. IRF2BP2 maintains macrophages in a non-inflammatory state and ablating IRF2BP2 in macrophages augments their inflammatory response (Chen et al., 2015). Chronic inflammation is a central driver of coronary artery disease (Almontashiri et al., 2013; Chen et al., 2015). Individuals homozygous for a 9-nucleotide deletion variant (rs3045215) in the 3′UTR of IRF2BP2 mRNA have increased risk of developing coronary artery disease. These individuals have reduced IRF2BP2 protein expression in their peripheral blood mononuclear cells (Chen et al., 2015), because the deletion variant disrupts a translational enhancer in the 3′UTR required for high levels of IRF2BP2 protein expression (Vilmundarson et al., 2021). Here, we showed that this deletion variant disrupts a stem-loop structure in the 3′ UTR sequences of IRF2BP2 mRNA that is needed to recruit the translation initiation factor eIF4H to maintain IRF2BP2 translation at high levels. This finding adds IRF2BP2 to the growing list of eIF4H-regulated mRNAs such as *c-myc*, *CIAP1*, *BCL-xL* (Vaysse et al., 2015).

While our finding is consistent with the requirement of eIF4H for enhanced translation of IRF2BP2, because we did not measure IRF2BP2 mRNA, we cannot exclude the possibility that eIF4H affects mRNA degradation. eIF4H is targeted by the herpes simplex virus (HSV) virion host shutoff (Vhs) protein (UL41), an endoribonuclease that degrades host cell mRNAs (Sarma et al., 2008). Knockdown of eIF4H prevents this mechanism from degrading a host mRNA (beta-actin), suggesting that some cellular mRNAs may be degraded by Vhs through the intermediary of eIF4H. However, in the absence of Vhs, eIF4H knockdown had no effect on beta-actin mRNA levels (Sarma et al., 2008), suggesting that eIF4H is not involved in stabilizing this mRNA under normal circumstances. While protein levels of IRF2BP2 were reduced after eIF4H knockdown, consistent with impaired translation, we cannot exclude the possibility that the stability of eIF4H-dependent mRNAs like IRF2BP2 is dependent on the presence of eIF4H. This could be addressed in future studies.

Macrophages exposed to the bacterial endotoxin LPS lower their baseline expression of IRF2BP2 to enable their full-blown inflammatory response to fight against infection (Martinez et al., 2006; Chen et al., 2015). The eIF4H-responsive stem-loop structure also mediates this LPS-induced downregulation of IRF2BP2 expression. We found that LPS promotes the recruitment of IRF2BP2 protein to these RNA sequences to suppress translation and the 9-nucleotide deletion disrupts this LPS-induced regulation. It is worth noting that our evidence for eIF4H interaction with the stem loop sequence is indirect, based on a differential response of the IRF2BP2 3′UTR-luciferase reporters to siRNA mediated knockdown of eIF4H. Similarly, we do not have direct evidence that IRF2BP2 binds to RNA, but rather is part of a cytoplasmic protein complex that shifts an RNA probe containing the non-deleted sequence at rs3045215 but not an RNA probe with the 9-nucleotide deletion. Also, while our experiments were carried out mostly in HEK293 cells, a similar regulation likely takes place in macrophages.

IRF2BP2 is predominantly found in the nucleus in macrophages, where it regulates the transcription of interferon-responsive genes (Chen et al., 2015). Our data here indicate that LPS increases the interaction of cytoplasmic IRF2BP2 to its 3′ UTR translational enhancer. Whether this occurs through an increased binding affinity of IRF2BP2 to its UTR sequences or to a protein that interacts with these sequences remains to be determined. Further, this may reflect increased nucleus-cytoplasmic shuttling of IRF2BP2. LPS was reported to drive cytoplasmic translocation of the RNA-binding protein Adenine-thymine (AT)-rich interactive domain 5a (Arid5a) in RAW264.7 macrophages (Higa et al., 2018). Whether LPS-induces IRF2BP2 nuclear export in macrophages is not known.

While IRF2BP2 is a known regulator of transcription, the findings here highlight a new role for IRF2BP2 as an RNA-associated protein that counteracts eIF4H-dependent translation of its own mRNA. Whether IRF2BP2 also affects translation of other eIF4H-dependent mRNAs awaits future studies.

It is also worth noting that IRF2BP2 mRNA and protein levels are not always correlated in cells other than macrophages and may reflect translational suppression by cytoplasmic export of IRF2BP2. Previously, we were puzzled by the fact that both cardiac and skeletal muscle express high levels of IRF2BP2 mRNA, but that IRF2BP2 protein levels are much lower in skeletal muscle compared to cardiac muscle (Teng et al., 2011). Unlike in cardiac muscle, IRF2BP2 protein is located predominantly in the cytoplasm of skeletal muscle upon differentiation (Teng et al., 2011), and may account for low IRF2BP2 protein levels, in view of the current finding of translational auto-suppression of IRF2BP2. Although the function of IRF2BP2 in cardiac and skeletal muscle is not fully elucidated, we reported previously that IRF2BP2 is induced by ischemia and may protect against oxidative stress in these tissues (Teng et al., 2010).

The protein isoform of IRF2BP2 encoded by the rs7545855 Ser^78^ polymorphism is co-inherited with the 3′UTR rs3045215 deletion variant. However, we could not identify a functional difference between the Ser^78^ and Pro^78^ isoforms, at least in terms of their suppression of translation via the 3′UTR eIF4H-responsive stem-loop sequences. This was contrary to our expectation given that phosphorylation at Ser^78^, in close proximity to the C4 zinc-finger domain required for homo- or hetero-dimerization with IRF2BP family members (Yeung et al., 2011), has been documented *in vivo* (https://www.phosphosite.org), a process that is precluded in the Pro^78^ variant. Thus, the Ser^78^ isoform co-inherited with the rs3045215 deletion variant might just be a “red herring.”

Eukaryotic translation initiation is facilitated by a “closed-loop” structure, where initiation factors (eIFs) connect the 5′cap and 3′poly(A) tail, enabling the recruitment of ribosomal subunits to initiate protein synthesis (Jacobson and Favreau, 1983; Dever, 2002). Regulatory sequences within the 5′UTR and 3′UTR recruit eIFs to control translation efficiency. Mutations within the regulatory elements of UTRs can alter translation efficiency and cause diseases. In patients with multiple myeloma, a C>T mutation in the *c-myc* internal ribosomal entry sequence (IRES) creates a stem-loop structure in the mRNA and increases translation of the *c-myc* protein 4.5 times compared to the wildtype (Cobbold et al., 2010). On the other hand, in patients with Charcot-Marie-Tooth disease, a C>T mutation in the 5′ IRES of connexin-32 creates a stem-loop structure that inhibits translation of the protein in Schwann cell (Hudder and Werner, 2000). While these examples are rare mutations occurring at the 5′UTR, to our knowledge, the rs3045215 deletion polymorphism is the first example of a common genetic variant in the 3′UTR that disrupts a stem-loop structure and suppresses translation.

Because IRF2BP2 is ubiquitously expressed and plays a key role in cell differentiation and proliferation pathways in both immune and non-immune cells, changes to its expression could affect many diseases, in addition to coronary artery disease. As such, homozygosity for the rs3045215 deletion allele affecting IRF2BP2 expression should be considered. For example, in acute myeloid leukemia cells, elevated IRF2BP2 expression promotes cancer cell survival and proliferation and suppresses the inflammatory NFKB pathway (Ellegast et al., 2022). Conversely, in medulloblastoma, elevated activity of IRF2BP2 suppresses PD-L1 expression making tumors more vulnerable to T-cells (Dorand et al., 2016). It would be interesting to learn whether the rs3045215 deletion allele influences outcomes in these two diseases. IRF2BP2 has also been implicated in hepatic inflammation and steatosis (Fang et al., 2020) and individuals homozygous for the rs3045215 deletion allele might be more vulnerable to obesity-associated hepatic steatosis.

The rs3045215 deletion variant is unique to humans and shows remarkable population-specific allele frequency differences. The deletion allele has a frequency of 13% in black Africans and 23% in Europeans but is the predominant allele in East Asians (74%) and Southeast Asians (55%). Given its potential to modulate the response to infectious diseases, this variant has likely undergone selection through generational exposure to different pathogens in different populations.

Taken together, our previous work and the present study propose that the deletion variant keeps macrophages in a chronic inflammatory state due to lower baseline expression of IRF2BP2 from the loss of the enhancing effect of eIF4H. On the other hand, this deletion variant would also impair LPS-induced auto-suppression of IRF2BP2 expression that is needed to maximize the inflammatory response to fight infection. These two conditions may account for increased risk of coronary artery disease and other macrophage-related chronic diseases (osteoporosis, inflammatory bowel disease) in people who carry 2 copies of the deletion variant.

## Data Availability

The original contributions presented in the study are included in the article/Supplementary Material, further inquiries can be directed to the corresponding authors.
