# Supplementary figures and images for: A 3′UTR polymorphism disrupts IRF2BP2 autoregulation through an eIF4H translational enhancer
Source: Front Genet. 2026 May 28;17:1846555. doi: 10.3389/fgene.2026.1846555 (PMC13252914; doi:10.3389/fgene.2026.1846555)

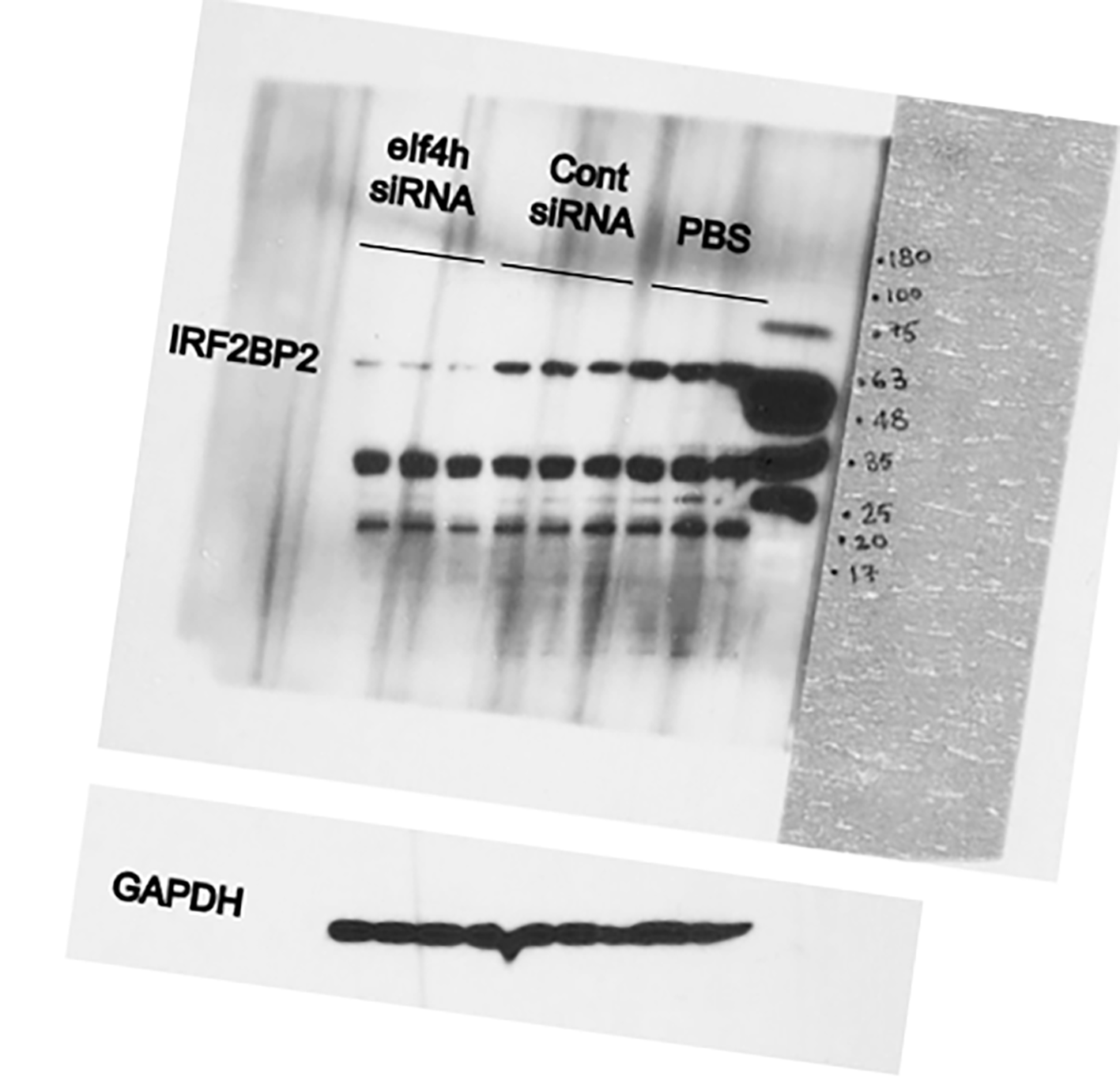

Supplement: Supplementary file 1 [file Image1.png]
